# Associations between metabolic rate and personality in a free-living small mammal are driven by date of birth

**DOI:** 10.1098/rsos.250801

**Published:** 2025-09-24

**Authors:** Jingyu Qiu, Carsten Schradin, Astolfo Mata, Neville Pillay, Heiko Rödel

**Affiliations:** ^1^ School of Animal, Plant and Environmental Sciences, University of the Witwatersrand Johannesburg, Johannesburg, South Africa; ^2^ Université de Strasbourg, CNRS, IPHC UMR 7178, 67000 Strasbourg, France; ^3^ Laboratoire d’Ethologie Expérimentale et Comparée UR 4443 (LEEC), Université Sorbonne Paris Nord, Villetaneuse, France

**Keywords:** behaviour, behavioural performance, life history, phenotypic correlation, stress response, metabolism

## Abstract

Physiological states are often regarded as drivers of personality differences, although the causal link between them remains unclear. Studies focusing on energy metabolism often report inconsistent associations with personality traits. We studied this association in 75 free-living female bush Karoo rats (*Otomys unisulcatus*) by testing their personality traits, resting metabolic rate (RMR) and, furthermore, metabolic rate stress response. We found a proactive behavioural syndrome, repeatable RMR and metabolic responses to an acute acoustic stressor. Linear mixed models showed that more proactive individuals exhibited higher RMR. To further explore this relationship, we conducted path analysis incorporating life history, environmental and ecological factors (such as date of birth, age, temperature and food abundance). This analysis revealed that the observed link between personality and RMR was not direct but instead may be mediated by the date of birth: individuals born later in the season were more proactive, had higher RMR and showed lower metabolic responses to acute stress. Importantly, personality and metabolic rate were not directly associated after accounting for date of birth. This finding highlights the importance of considering broader ecological/life-history contexts when interpreting physiological–behavioural correlations and offers a possible explanation for previous contradictory results regarding the personality–RMR relationship.

## 1. Introduction

From fundamental biological processes to complex behaviours, energy fuels every function of living organisms [1]. Consistent individual differences in behaviour, or animal personality [2,3], have been hypothesized to correlate with individual differences in energy expenditure or metabolic rate (MR) [4–6]. One commonly tested prediction is that bolder, more active and more explorative individuals have a higher resting MR (RMR) than shy, inactive individuals, as such behaviours may be linked to higher energy demands and promote energy acquisition [7–9]. For instance, increased time spent in exploration and foraging may improve access to food and other resources, by providing greater net energy gain to sustain a generally higher level of metabolism. Previous studies have investigated the link between individual differences in energy cost and personality traits in different taxa, such as in fish [10,11], birds [12,13] and mammals [14,15] including in humans [16]. However, these studies have revealed widely inconsistent results, as more than half of such studies, as reviewed in [7], report positive correlations between behaviour and energy metabolism, while the remaining ones found no or negative correlations. These discrepancies highlight the need to better understand the role of external factors, such as those related to ecology or life history, in shaping the relationship between personality traits and RMR.

Efforts have been made to explain the mechanisms underlying possible associations between behaviour and energy metabolism. Because both traits influence energy intake and expenditure, different models have been proposed to predict how behaviour and energy metabolism interact, and these have been repeatedly tested in empirical studies [4–7]. The ‘performance model’ assumes that higher MRs reflect a greater capacity to mobilize energy and also predicts that more active individuals will have higher RMR [6,7]. This model aligns with the frequently found positive association between RMR and more proactive (bolder, more active and exploratory) personality types [5,7–9]. Alternatively, the ‘allocation model’ proposes that energy costs in behaviour and basal metabolism are constrained by a shared energy budget, predicting that more active individuals will have lower RMR. The ‘independent model’ assumes that energy costs associated with behaviour and basal metabolism are independent, and therefore no relationship is predicted. Although the ‘performance model’ appears to be favoured by a large number of studies, as reviewed in [5,7], further empirical data on the possible relationships between metabolism and behaviour are needed, particularly in wild populations [17].

When exploring causal links between metabolism and behaviour, it is important to recognize that both MR and personality are shaped by ecological conditions and life-history factors. For example, early life environments can have long-lasting effects on personality. In young laboratory rats (*Rattus norvegicus*) around the time of weaning, heavier individuals were bolder and more exploratory, and litter size influenced behavioural expression of anxiety [18]. In zebra finches (*Taeniopygia castanotis*), nutritional restriction early in life affected physiological traits, with individuals experiencing nutritional restrictions as nestlings exhibiting higher basal MRs in adulthood [19], indicating long-lasting physiological effects of early life experiences. Thus, individual personality and RMR may be concomitantly influenced by external factors, especially during early development, resulting in statistically significant correlations that are not necessarily causal. In other words, both traits may respond to the same underlying factor(s), producing a potentially spurious association between them. Identifying and accounting for these shared influences is essential in understanding the behaviour–RMR relationship that varies across species and ecological contexts [7]. Individual-based studies on wild animals, combined with multifactorial statistical approaches such as path analysis, offer a powerful framework to identify causal pathways in multi-correlational relationships between behaviour, physiology and ecological contexts [20,21]

In seasonally breeding mammals, the date of birth within the reproductive season influences early life conditions, leading to divergence in individual life-history trajectories [22,23] as well as in personality [24]. Early borns may experience lower population densities, less intense resource competition and have the opportunity to reproduce within the season they were born. Under the pace-of-life framework, divergence in early life conditions can lead to differential trade-offs between survival and reproduction, which manifest as differences in life history along the behavioural and physiological traits continuum [25,26]. Accordingly, early borns are expected to adopt a ‘fast’ pace of life, having a proactive (more active, explorative and bold) personality and a higher MR. However, the predicted link may be disrupted by ecological constraints; for example, in our study species, the bush Karoo rat (*Otomys unisulcatus*), individuals born later in the season display more proactive personality types, likely due to intense resource competition and contrary to suggestions of the pace-of-life framework [21]. However, whether their metabolic variables correspond to this framework remains unknown. The birth date within the breeding season could be an important factor influencing or even driving the association between personality and MR; however, there is still a lack of studies that consider birth date [7,27].

Due to inconsistent findings regarding the association between personality and RMR/total energy expenditure, it has been suggested that complementary aspects of energy metabolism, such as the metabolic response to acute stress, may serve as an informative physiological predictor to test the correlation with personality [15]. The acute physiological stress response enables immediate behavioural responses to critical situations [28]. In vertebrates, acute stressors such as a sudden encounter with a predator or other risky situations trigger the sympathetic stress response to immediately activate energy supplies [29]. Proactive (i.e. bolder, more active and more exploratory [30]) and reactive animals (with contrasting personality traits, respectively) tend to exhibit divergent stress responses [31], with the acute sympathetic stress response tending to be more pronounced in proactive individuals [30,32]. These differences might affect the dynamics of metabolic responses in animals with varying personality types. It is surprising that the relationship between the direct, short-term metabolic consequences in response to stress in different personality types has so far hardly been studied (but see [10]).

In this study, we used the free-living bush Karoo rats as a model to evaluate the correlation between personality traits, RMR and the metabolic response to acute stress, as well as whether these factors are associated with individual birth dates. The bush Karoo rat is a diurnal, small (adult body mass approx. 100 g), solitary rodent [33] that lives in the semi-arid Succulent Karoo of South Africa. It experiences a seasonal environment with superabundant food in the moist spring, followed by the very hot and dry summer with low food availability. A previous study revealed that bush Karoo rats exhibit distinct personality differences [34], influenced by their date of birth: later-borns that experience higher population density and lower resource availability were more proactive (bolder, more active and explorative) than earlier-borns [24]. Therefore, we hypothesize that (i) RMR is higher in bolder, more active and more exploratory individuals and that (ii) such more proactive individuals show a higher MR in response to an acute stressor. (iii) We also hypothesize that date of birth is positively correlated with personality and with energy metabolism, with later-born individuals being more proactive, having higher RMR and showing a higher MR stress response. We used multifactorial path analysis to disentangle potential causal processes [20] and (iv) hypothesize that the association between personality and MR is not direct, but is instead likely driven by their shared relationship with date of birth.

## 2. Material and methods

### 2.1. Study species and study site

Our study was conducted in the Goegap Nature Reserve, situated in the Northern Cape province of South Africa, located within the Succulent Karoo biome, a semi-arid region recognized as a biodiversity hotspot [35]. The field site receives an annual rainfall of 160 mm on average, and temperatures vary from −1.5 to 24°C during winter and from 4 to 42°C during summer (data from a weather station at the field site). Most of the rainfall occurs in autumn and winter, leading to abundant ground vegetation that supports the onset of reproduction in bush Karoo rats [33]. In our study population, parturitions usually occur between mid-July and late November [24].

### 2.2. Study area and trapping

We collected data at a study site of approximately 4.5 hectares from August 2022 to December 2023. Bush Karoo rats take refuge in ‘stick lodges’, which they construct from dry plant materials and can be up to 0.7 m high [36–38], making them easy to locate in the field.

Daily trapping was done to mark and track the study population. We used Sherman traps and locally produced metal traps (26 × 9 × 9 cm) of the Sherman trap style. Traps were set at lodge entrances before sunrise and checked every 30 min. All traps were closed within 2 h after sunrise (i.e. trapping was stopped) to prevent the animals from overheating. The field site was divided into six areas. Daily trapping was always carried out at only two of these areas at the same time for three days, before moving on to the next two areas. Trapping and marking were carried out at all occupied stick lodges throughout the year as part of our long-term data collection. Therefore, focal individuals for this study could be identified by their aluminium band ear tags carrying a unique individual number (National Band and Tag Co., Newport, KY, USA).

### 2.3. Timing of experimental procedures

Additional trapping of focal individuals for this study was done by J.Q. and research assistants. Captured focal individuals were transported inside the trap to a field laboratory, which was less than a 10 min walking distance from the field site. The study focused exclusively on females, the more philopatric sex in this species. During one morning, we measured behavioural responses (in standardized tests) and MRs in up to four rats at a time (see details below). Thereafter, individuals were released at their lodge 3−6 h after capture. Except when checking their identity, performing behavioural tests and MR measurements, the rats remained in the trap until they were released. This was done because they were accustomed to the traps and showed no obvious signs of distress when inside, compared to keeping them in unfamiliar transparent holding cages.

Rats were scheduled for four repeated (behavioural and metabolic) tests at two age classes. The first behavioural and MR tests were scheduled at an age of approximately six weeks, the youngest age when bush Karoo rats reach sexual maturity [39]. A second test was scheduled approximately two weeks later. In the first year of study, individuals that survived long enough were re-tested at an older age at approximately twenty weeks (third test) and again two weeks later (fourth test). Due to logistical constraints in the second year of study, focal individuals only underwent up to two tests at an age of approximately six weeks and again two weeks after. Trapping of focal individuals was not always successful, so tests and measurements were sometimes delayed or not carried out due to the temporary or permanent disappearance of the individuals.

Note that the focal animals used in this study were part of a larger sample, in which we previously demonstrated that individuals born later in the breeding season tend to exhibit more proactive personality traits [24].

### 2.4. Calculation of birth date

We calculated the date of birth as the date of first capture minus the age (in days) at this first capture. The age at first capture was estimated using the individual body mass (*M*) at the early postnatal stage (at first trapping), based on the following Gompertz equation reported for bush Karoo rats in [39]:


$$\text{Age at first capture (in days)}=\frac{\ln\left(\ln\left(\frac{88}{M}\right)\right)}{-0.052}+15.$$

This equation describes a strong and predictable relationship between body mass and age during early development of bush Karoo rats (*r*^2^ = 0.85, *p* < 0.001) [39]. In our study, we considered individuals only with a body mass of up to 65 g at first capture, corresponding to a predicted age of 38 days, as the association between body mass and age was almost linear within this range [39].

Individual growth during juvenile life can also be affected, however, by factors such as prevailing environmental conditions and food availability [40], possibly leading to differences in the association between juvenile body mass and age among seasons and years. Thus, it cannot be entirely ruled out that estimating an individual’s exact date of birth based on growth patterns across different seasons and years may introduce a misleading sense of precision. To address this potential pseudo-precision, we also employed a more conservative approach by estimating birth dates in two-week and four-week intervals, rather than daily. Using these coarser estimations, we re-ran all key analyses of our study and obtained consistent results (see electronic supplementary material, tables S4 and S5), underscoring the robustness of our findings.

### 2.5. Personality tests

Boldness, activity and exploration were measured using the starting box (SB) and open field (OF) tests (more details in [24]). The test arena was a chamber (100 cm long, 85 cm wide and 65 cm high) made of white coated plywood panels, with two liftable doors symmetrically located on the two short sides of the rectangular arena (see fig. 1 in [24]). For testing, rats were individually introduced into the SB, which was a 10 cm^3^ black acrylic and opaque squared box connected to the arena. The SB was separated from the test arena by a closed door.

#### 2.5.1. Starting box test

After the focal individual rested for 3 min in the SB (habituation time), the door to the test arena was lifted, and we recorded the behavioural variable for boldness, as quantified by the probability of entering the arena, and defined as whether or not (1/0) the animal entered the test arena in the following 10 min.

#### 2.5.2. Open field test

The OF test began once the rat had entered the arena. Thereafter, the door was closed to prevent it from returning into the SB. The behaviour of the focal individual in the OF was video recorded for 5 min. Considering the experimental setting and the species’ behavioural pattern [41], we quantified behavioural variables that are biologically meaningful for bush Karoo rats. Activity was measured by the total distance travelled, defined as the total distance of locomotion in the arena measured in centimetres. Exploration was measured by the % time of exploration, defined as the percentage of time the animal spent exploring walls and corners by sniffing or putting the front paws against the walls.

### 2.6. Metabolic rate measurements

After the behavioural tests, individuals were transferred back to their original traps to rest for 2−3 h. Respirometry measurements were obtained after the behavioural test of the last individual of the day, at least 4 h after sunrise, which corresponded to the non-active period of bush Karoo rats [42].

MR was measured by oxygen consumption using a flow-through system with excurrent flow. The system included an RH-300 water vapour analyser (Sable Systems International, SSI, Las Vegas, Nevada), a FoxBox respirometer (SSI, Las Vegas, Nevada) and one metabolic test chamber and two channels (one for the test chamber and one for baseline). The baseline channel was an air tube directly connected to ambient air, while the test channel included a 1 l test chamber connected to ambient air. A three-way valve was used to control the airflow, directing it either into the test channels with the focal individual or into the empty baseline channels for baseline recordings.

Ambient air was drawn from outdoors at a flow rate of around 700 ml min^−1^. It first passed through a spiral copper tube to have full thermal exchange to stabilize the air flow temperature at 30°C (Pelt 5, SSI, Las Vegas, Nevada). The air then flows through either the test chamber with the focal animal inside (test channel) or an empty tube (baseline channel), then passes through the RH-300 (water vapour pressure) and finally the FoxBox respirometer (carbon dioxide and oxygen concentration) before being released back to the ambient air. Data were recorded and analysed using the ExpeData software package (SSI, Las Vegas, Nevada). The system undergoes regular calibration (see detailed descriptions in electronic supplementary material, table S1, and in the FoxBox user manual). Individual O_2_ consumption (ml h⁻¹, for later analysis corrected by gram body mass⁰·⁷⁵, see details below) was calculated using the equations for the pull system [43] (electronic supplementary material, table S1). During the MR measurements, we monitored the animal with a webcam (C170, Logitech) connected to a computer situated outside the test area. After the MR measurements, the focal individuals were given succulent plants and bait (a mixture of bran flakes, salt and sunflower oil) as compensation for missed foraging opportunities, before being released back to the stick lodge from where they had been captured.

Rodents are sensitive to threat signals, such as auditory cues [44,45]. Based on this sensory capacity, we measured individual metabolic responses to an acoustic stressor. A key finder (Thousandshores Deutschland GmbH, model HL-KF02A, manufacturer model KF02A, 5 × 2 × 0.5 mm) controlled by a remote was placed in advance inside the test chamber, separated from the focal individual by a honeycomb metal plate. Pressing the remote control produced a 10 s alarm, consisting of a sequence of high-frequency short beeps of around 90 dB. The alarm sound was assumed to induce acute stress in the animals. This was confirmed by our preliminary experiments and direct observation, verifying that bush Karoo rats showed either a startle response and/or freezing behaviour to this acute acoustic stressor.

The metabolic measurement started with a 5 min baseline recording. During this time, the baseline channel was connected, and the focal individual was introduced into the test chamber for later testing. Thereafter, the airflow was switched to the test channel for at least 20 min. The first 10 min were discarded for RMR calculation because airflow had not yet reached equilibrium. If the individual was predominantly restless during the following 10 min of measurement, the measurement time was extended until the individual entered a resting state for at least 1 min. Resting state was determined by surveillance via the webcam monitor and by the readings of the FoxBox, through which we verified that the individual stopped any locomotor activity and that the reading of O_2_ concentration remained stable over time. Considering that keeping wild individuals in the laboratory for too long could negatively impact their welfare, we chose to record MRs at high frequency (i.e. one sample per second) over a at least 10 min period (after 2−3 h resting in the trap and a preceding 15 min acclimatization period in the chamber) as a practical and ethical compromise to shorten the measurement duration. In our focal individuals, this sampling strategy was sufficient to capture the low, stable 30 s sections of data suitable for calculating RMR.

After RMR measurement, and while the focal individual remained at rest, the remote control of the key finder was triggered, producing an acute auditory stimulus (alarm) for 10 s. The 10 min recording following the alarm was used for the calculation of metabolic response to acute stress. When the focal individual resumed a resting state, the alarm sound was triggered again to repeat the measurement.

Individual RMR was retained as the mean O_2_ consumption during the lowest 30 s time span during the last 10 min before the first alarm. RMR used in the following analysis was corrected for body mass according to Kleiber’s law, using the formula RMR = individual RMR/body mass⁰·⁷⁵ [46].

The metabolic response to this acute stressor was measured using two variables: (i) maximum MR stress response, calculated as the maximum O_2_ consumption (per hour and gram^0.75^) within 1 min after the alarm sound, divided by the O_2_ consumption at resting state; and (ii) integral MR stress response, calculated as the mean integral of O_2_ consumption (per hour and gram^0.75^) during 1 min after the acoustic stimulus, divided by the total O_2_ consumption in 1 min at resting state. For both variables, the mean of the two repetitions was used for statistical analysis.

### 2.7. Statistical analyses

For each individual, we included data only from test sessions during which we managed to collect both MR and behaviour data. In total, 75 females were tested 148 times during the 1st, 2nd, 3rd and 4th tests, among which 56 females were tested 82 times during the 1st and 2nd tests (34−100 days old, mean of 55 days), and 38 females were tested 66 times during the 3rd and 4th tests (138−213 days old, mean of 150 days). Missing data were due to individuals disappearing over the time of study, as newborn bush Karoo rats in this field site experience high mortality rates. Statistical analyses were carried out using R software, v. 4.4.2 [47].

#### 2.7.1. Repeatability

Repeatability (consistency over time) of behavioural and metabolic variables was analysed using generalized linear-mixed-model (GLMM)-based intra-class correlations, including individual identity as a random intercept factor, using the R package *rptR* [48]. We always assumed a Gaussian distribution except for the behavioural variable ‘probability to enter the arena’, which followed a binomial distribution (table 1). Repeatability was measured as short-term repeatability (1st and 2nd test sessions) when the individuals were still young adults and as long-term repeatability (1st to 4th test sessions), including both age classes. Short-term repeatabilities between the 3rd and 4th test sessions were also tested, and results are given in electronic supplementary material, table S2. Values of *p* were calculated using Monte Carlo permutation tests (10 000 permutations of the model) implemented in the package *rptR*.

**Table 1.** Repeatability estimation (*R*, including its 95% confidence interval CI_95%_) of behavioural variables measured in repeated SB and OF tests.

	(a) short-term repeatability			(b) long-term repeatability		
	*R*	CI_95%_	*p*	*R*	CI_95%_	*p*
probability to enter the arena (SB)^a^	0.980	(0.985, 0.999)	**0.025**	0.512	(0.150, 0.986)	**<0.001**
distance travelled (OF)^b^	0.481	(0.127, 0.731)	**0.009**	0.323	(0.120, 0.571)	**0.003**
% time exploration (OF)^b^	0.576	(0.275, 0.779)	**0.002**	0.378	(0.158, 0.559)	**<0.001**

Analysis of data from 75 female bush Karoo rats by GLMM-based intra-class correlations (^a^binomial distribution, ^b^Gaussian distribution), including individual identity as a random intercept factor and number of tests the individual has experienced before as fixed variance. (a) Short-term repeatability was based on (up to) two behavioural tests during the young adult stage (56 individuals, *n* = 82 measurements for each behavioural variable during around postnatal weeks 6−8). (b) Long-term repeatability was based on all (up to) four behavioural tests of all age classes (measurements during approximately postnatal weeks 6−8 and postnatal weeks 20−22), i.e. from young adult stage to older adult stage (75 individuals, *n* = 148 measurements for each behavioural variable). Values of *p* were calculated by permutation tests (10 000 Monte Carlo permutations); significant effects (*p* < 0.05) are highlighted in bold. Further details are provided in electronic supplementary material, table S2.

^a^ Binomial distribution.

^b^ Gaussian distribution.

All behavioural and metabolic variables (presented in tables 1 and 2) were standardized (scaled), and some of them were transformed for analysis (see below) in the same way as for the path analysis. Our previous study [24], for which we used a larger dataset (including the data analysed in the current paper), found significant differences over time regarding the behavioural measurements. That is, individuals showed a generally higher level of behaviour in the first test session than in the subsequent ones, possibly due to familiarization effects to the setting [24]. Such strong population-level differences over time (over consecutive test sessions) with respect to the animals’ behavioural responses, as well as their MRs, can potentially mask the detection of individual-based repeatability. Thus, we included the number of tests the individual has experienced before (0/1/2/3) as a fixed factor to control for the possible influence of such differences among test sessions.

**Table 2.** Repeatability estimation (*R*, including its 95% confidence interval CI_95%_) of three metabolic variables.

	(a) short-term repeatability			(b) long-term repeatability		
	*R*	CI_95%_	*p*	*R*	CI_95%_	*p*
RMR	0.398	(0.049, 0.669)	**0.026**	0.393	(0.184, 0.574)	**<0.001**
maximum MR stress response	0.367	(<0.001, 0.675)	**0.045**	0.097	(<0.001, 0.307)	0.180
integral MR stress response	<0.001	(<0.001, 0.364)	>0.999	0.013	(<0.001, 0.324)	0.462

Analysis of data from 75 female bush Karoo rats by LMM-based intra-class correlations (for Gaussian distribution), including individual identity as a random intercept factor and the number of previous tests as fixed variance. (a) Calculations on short-term repeatabilities were based on data from (up to) two behavioural tests during the young adult stage (56 individuals, *n* = 82 measurements for each behavioural variable during around postnatal weeks 6−8). (b) Long-term repeatabilities were based on data from all (up to) four behavioural tests of all age classes (measurements during around postnatal weeks 6−8 and postnatal weeks 20−22), i.e. from young adult stage to older adult stage (75 individuals, *n* = 148 measurements for each behavioural variable). Values of *p* were calculated by permutation tests (10 000 Monte Carlo permutations); significant effects (*p* < 0.05) are highlighted in bold. See more details in electronic supplementary material, table S2.

#### 2.7.2. Association between behavioural traits and metabolic rate

We first tested for direct associations between the behavioural variables (three predictors: probability to enter the arena; distance travelled; % time exploration) and MR variables (three response variables, used in separate models: RMR; maximum MR stress response; integral MR stress response). To establish whether there was a direct relationship, we explicitly did not include any further predictor variables, such as the individual date of birth, in these models. This was done by linear mixed-effects models (LMMs) using the R package *nlme* [49]. Due to collinearities between the three behavioural variables, they were considered separately in different models, i.e. nine models were calculated in total (table 3). All models included individual identity as a random intercept factor.

**Table 3.** Associations between behavioural traits and MR variables measured in repeated SB and OF tests.

	(a) RMR		(b) maximum MR stress response		(c) integral MR stress response	
	*β ±* SE	*p*	*β ±* SE	*p*	*β ±* SE	*p*
probability to enter the arena (SB)	−0.027 ± 0.189	0.884	0.170 ± 0.191	0.370	−0.012 ± 0.189	0.950
distance travelled (OF)	**0.171 ± 0.075**	**0.027**	−0.072 ± 0.083	0.391	−0.114 ± 0.082	0.170
% time exploration (OF)	**0.228 ± 0.103**	**0.029**	−0.099 ± 0.083	0.236	−0.006 ± 0.006	0.302

**Figure 1.**
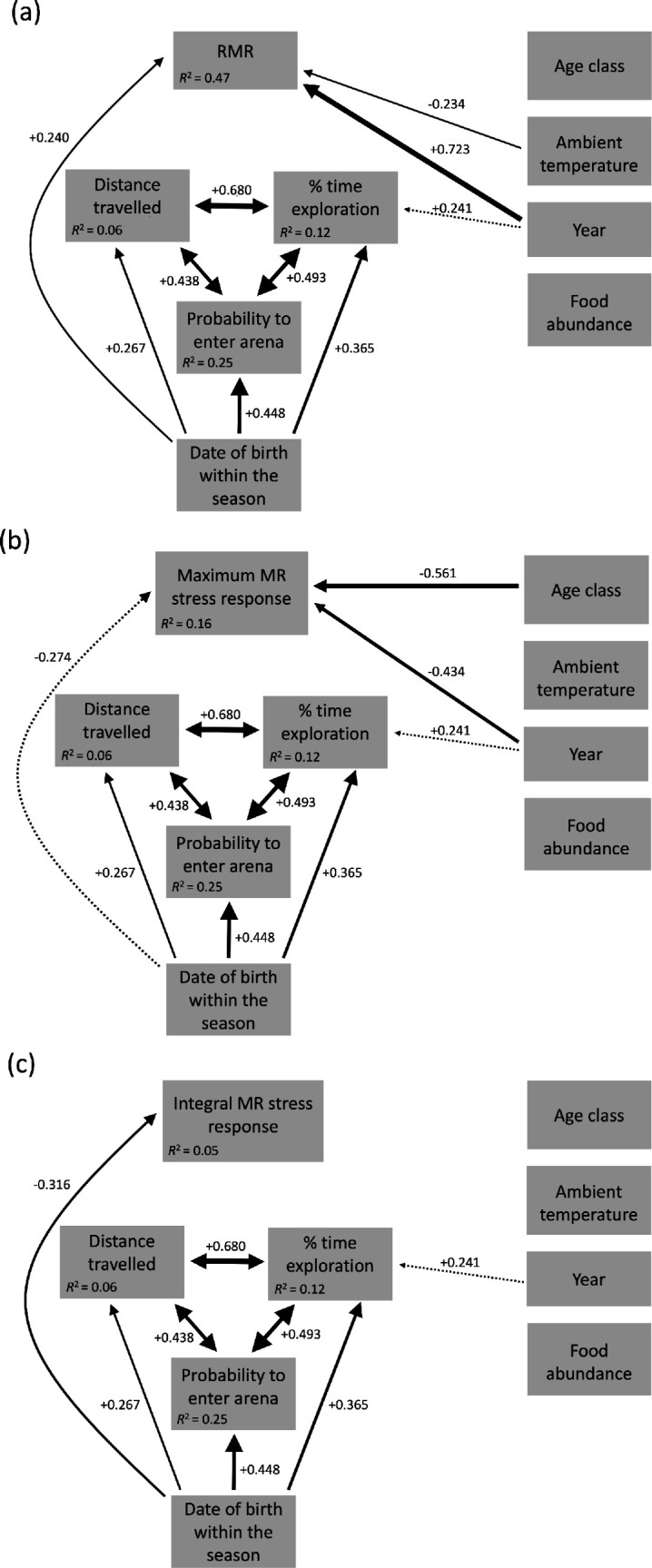
Path models for the effect of date of birth, personality traits and other variables on MR variables in the bush Karoo rat. Response variables in the different path models were (a) RMR (ml O_2_ per h and g^0.75^ body mass), (b) the maximal MR stress response (%, within 1 min) and (c) the integral MR stress response (%, within 1 min); all models including year (2022, 2023), individual age class (young or old), ambient temperature and food abundance as fixed variables (see electronic supplementary material, table S1, for details). Standardized estimates are given next to the arrows connecting the different variables; arrow thickness reflects the absolute value of the standardized estimates. Solid arrows represent significant associations (*p* < 0.05), and dashed arrows indicate statistical tendencies (*p* < 0.10); non-significant associations (*p* > 0.10) are not depicted. The two-sided arrows between the different behavioural variables indicate collinearities among them, which were implemented into the path models as correlated errors. Marginal *R*^2^ (given in the grey boxes) summarize the effects of all predictor variables towards a variable. We also tested for potential allometric effects of body mass at testing on the different metabolic variables (a–c), and none of these associations were statistically significant (all *p* > 0.70). More details on statistics are given in electronic supplementary material, table S3.

Analysis of data from 75 female bush Karoo rats by LMM with one MR parameter as dependent variable per model. Due to collinearities between the three behavioural variables (figure 1), three separate models were calculated for each personality trait as predictors (covariates), all including individual identity as a random intercept factor. Standardized estimates (*β*) with standard errors are given. Values of *p* were calculated by permutation tests (10 000 Monte Carlo permutations); significant effects (*p* < 0.05) are highlighted in bold.

Values of *p* were calculated using Monte Carlo permutation tests (10 000 permutations) in the *pgirmess* package [50]. Even though permutation tests do not rely on a normal distribution of model residuals [51], we verified whether variances were homogeneous for all models by plotting fitted values versus the residuals [52], since unequal variances in permutation testing can inflate type I error rates [53]. Thus, we homogenized model variances by applying transformations to two of the MR variables: the response variable, maximum MR stress response, was square-root transformed after adding a value of 9.76 to obtain positive values, and the integral MR stress response variable was log-transformed after positivizing all values by adding 25.07. These transformations of these two MR variables were also used later in the LMM-based path analysis, as described below.

#### 2.7.3. Path analysis

We examined the chain of relationships among date of birth, behavioural traits and metabolic variables, considering further possible predictors using path analysis [20]. The date of birth can influence behavioural traits in the bush Karoo rat [24], and we aimed to test whether the date of birth would directly and/or indirectly (via its effects on behavioural traits) affect the MR variables. In other words, we aimed to assess whether the date of birth affected the variation of both individual behavioural traits as well as metabolic variables.

Path analyses were carried out with the R package *piecewiseSEM* [54], based on combinations of LMMs and a GLMM for binomial data with a logit link. The latter was used for modelling effects on the binary behavioural response variable ‘probability to enter the test arena’ (see details on the structure of the complete path model in electronic supplementary material, table S3). LMM and GLMM were calculated using the R functions *lmer* and *glmer* (package *lme4* [55], linked to the package *piecewiseSEM* [54]), including individual identity and the number of tests the individual has experienced before (0/1/2/3) as random intercept factors. We calculated separate path models for the three response variables: RMR, maximum MR stress response and integral MR stress response. Initially, we constructed directed acyclic graphs to examine the causal relationships of each of the metabolic variables with the three personality traits (probability to enter the arena, distance travelled and time exploration), the individual date of birth within the season, the year of testing (2022/2023), age class at testing (young adult/older adult), ambient temperature (°C) and food abundance (number of food plants per 4 m^2^ [56,57]). We also accounted for potential allometric effects of the body mass at testing on the different metabolic variables, because smaller individuals frequently show a higher RMR per unit body mass [58,59]. Details are in figure 1a–c and in electronic supplementary material, table S3.

Because path analyses, as implemented in the R package *piecewiseSEM*, use only covariates, the 2-level factors, age class and year, were transformed into numerical variables and included the values −1 and +1 [54]. The expected collinearities between the three different behavioural variables (see [24]) were implemented into the path models as correlated errors [54] (see electronic supplementary material, table S3). Alternatively, we also constructed path models with inverted purported causal direction between personality traits and the different measures of MR. That is, we set the three personality traits instead of one of the measures of MR as the top tier in our directed acyclic graph (see figure 1a–c versus electronic supplementary material, figure S3a–c). However, the analysis of both versions of directed acyclic graphs revealed the same results (details in electronic supplementary material, tables S3 and S6–S8).

All continuous variables were scaled for analysis to obtain standardized slopes (*β*), where the absolute value of the standardized slope (beta weight) can be interpreted as the relative effect size of an association [54]. This was done by using the scale function, which is part of the base package in R [47]. Specifically, for each continuous variable, we centred the data by subtracting the mean from each value and scaled them by the standard deviation, so that all values are expressed as differences in standard deviations from the mean. Such scaling of continuous variables was also done for all the above-mentioned analyses. For each response variable in the path diagram, we also report (multiple) marginal *R*^2^ (figure 1), which represents the proportional explained variance excluding the contribution of random factors [60]. For all LMMs, we checked for the normal distribution of model residuals verified by visually checking normal probability plots and assessed homogeneity of variances by plotting residuals versus fitted values [52]. We verified the goodness of fit of the global path models using chi-squared tests [54].

## 3. Results

### 3.1. Consistent individual differences in behaviour

We found significant repeatability over time for all three behavioural variables in the short term as well as the long term (table 1). Inter-individual differences in these behaviours remained consistent both during the repeated measurements within around two weeks of time in young adults (1st and 2nd tests; table 1a), as well as at the long term over different age classes, i.e. in young and older adults (all four test sessions; table 1b). Significant short-term repeatabilities for the probability to enter the arena and the time spent exploring the OF during the older adult stage are available in electronic supplementary material, table S2.

### 3.2. Consistent individual difference in measures of metabolic rate

We also found significant repeatability over time for RMR, both at the short term in young adults (1st and 2nd tests; table 2a) and at the long term (table 2b).

The repeatability of the maximum MR stress response was significant at the short term, i.e. between two test sessions for two weeks as young adults (table 2a). No significant repeatability or tendencies of such were found for the integral MR stress response.

When testing short-term analyses during the older adult stage, we did not find significant short-term repeatabilities for any metabolic parameter (electronic supplementary material, table S2).

### 3.3. Associations between personality traits and variables of metabolic rate

When using correlative analyses (by LMM accounting for repeated measurements), without controlling for any potentially confounding variables, RMR was significantly and positively associated with the two behavioural variables measured in the OF test: that is, individuals with higher RMR travelled longer distances and explored longer in the OF (table 3a).

**Figure 2.**
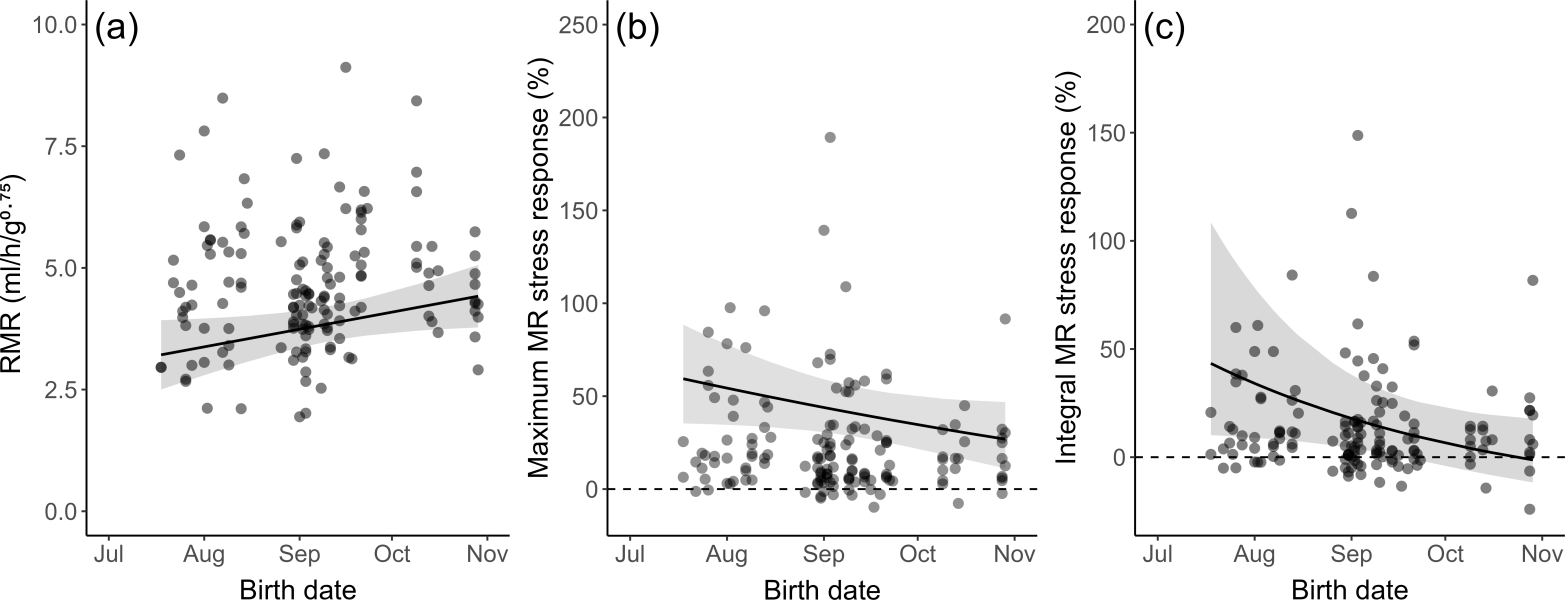
Effects of birth date within the breeding season on MR variables in the bush Karoo rat. All effects presented here are statistically significant (see statistics in figure 1a–c). Regression lines (including 95% confidence intervals given as grey shadings) are based on parameter estimates obtained by a path analysis based on combined LMM and GLMM; regression lines of (b,c) are based on back-transformed parameter estimates (see details in electronic supplementary material, table S3).

Note that for most individuals, we found a rather clear, short-term fluctuation (mostly increase) in MRs after the playback of the acoustic stressor, as evident by the mostly positive values of the maximal MR stress response and integral MR stress response (figure 2b,c). However, we did not find any significant associations of these two metabolic variables with any of the three behavioural variables (table 3b,c).

### 3.4. Path analysis of the association between date of birth, personality and metabolic rate

Our path analyses confirmed significant associations between all three behavioural traits, thus forming a behavioural syndrome. Individuals who were more likely to enter the open arena also travelled a larger distance in the arena and spent more time exploring the arena. All three behavioural traits were significantly associated with the individual date of birth. As already shown in our previous study [24], later-born individuals were more exploratory (*β* = 0.365), travelled a greater distance in the OF (*β* = 0.267) and also entered the open arena with a higher probability within the 10 min of testing (*β* = 0.448; figure 1; details in electronic supplementary material, table S3).

We found direct and significant effects of the date of birth within the breeding season on RMR and integral MR stress response, as well as a tendency for the maximum MR stress response, although with different directions. Later-borns had a significantly higher RMR (*β* = 0.240; figures 1a and 2a), a lower integral MR stress response (*β* = −0.316; figures 1b and 2b) and tended to have a lower maximum MR stress response (*β* = −0.274, *p* = 0.072; figures 1c and 2c). However, there were no significant and direct associations between any of the behavioural variables and the animals’ RMR (figure 1a), their maximum MR stress response (figure 1b) or their integral MR stress response (figure 1c). These results remained the same when decreasing the accuracy of our calculation of the date of birth from a daily scale to two-week intervals or even to four-week intervals (electronic supplementary material, tables S4 and S5). Furthermore, we found the same results when inversing the purported causal direction between personality traits and MR variables in our path analyses (electronic supplementary material, figure S3a–c and tables S6–S8).

We also found some significant effects of other confounding variables on the three MR variables. RMR decreased significantly with increasing ambient temperature (*β* = −0.234; figure 1a; electronic supplementary material, figure S3). There were also significant differences between both years of study; RMR (*β* = 0.723) measured during the second year was higher than during the first year (figure 1a and electronic supplementary material, figure S3). Food abundance had no significant effect on any of the behavioural or metabolic variables tested (figure 1a–c).

## 4. Discussion

We studied whether the date of birth within the breeding season could lead to a positive but non-causal correlation between RMR and personality in female bush Karoo rats. In support of this, in our first analysis, which did not account for date of birth nor other ecological factors, we found that RMR and personality were significantly correlated. However, when applying a path analysis that considered additional parameters, we identified the date of birth as the likely underlying factor of this significant correlation, influencing both personality and RMR without there being any direct link between the two.

Our study (see also [24]) confirms the existence of personality types in wild rodents under natural conditions [61–64]. In addition, we provided field evidence for the repeatability of RMR at the individual level, i.e. individual RMR values tended to remain relatively consistent over short (2 weeks) and even over longer time spans based on up to four repeated measurements during approximately 14 weeks. Although repeatability of metabolic traits has been widely studied in laboratory settings, evidence from free-living populations remains limited [65,66], and our findings help to address this gap.

Variation in RMR between individuals has been assumed to explain individual differences in personality, but results from previous studies were inconsistent [5,7]. This suggests that additional factors might be important mediators in this relationship [7]. The findings of our first correlational analysis between behaviour and MR, which we deliberately carried out without considering any external, potentially confounding factors, suggested a positive relationship between RMR and proactive personality traits. However, such significant associations between RMR and personality traits disappeared when further external parameters were considered in our path analysis (see figure 1a–c and electronic supplementary material, figure S3a–c, for an alternative analysis). External factors might independently influence both personality and RMR, as shown for predation pressure in a study on great tits (*Parus major*) [13], and here for the date of birth. Our previous research suggests the availability of stick lodges, which offer protection to bush Karoo rats from extreme climatic conditions, is likely the main driver of proactive personality in later-born individuals [21]. This may explain the link between birth date and RMR within the pace-of-life framework. During the late breeding season, the availability of stick lodges decreases as population density increases. Consequently, later-born individuals may benefit from adopting a ‘fast’ pace-of-life, characterized by a more active, exploratory, bold personality and higher MR, thus enhancing their ability to secure these essential resources [5,6].

Returning to the conceptual framework proposed to explain different ways that metabolism can be linked to personality [4,6,7]: at first glance, the positive—although, as we argue, non-causal—correlation between proactive personality and RMR appears consistent with the ‘performance model’. As reviewed in [4], this model predicts a positive relationship between RMR and proactive personality traits, because such individuals are expected to sustain a generally higher energy throughput, possibly due to their relatively higher proportion of lean muscle mass that contributes to elevated metabolic activity [67]. Examples of this positive association between RMR and proactive personality traits have been shown in several species across various vertebrate taxa, including different species of small mammals (reviewed in [5,7]). Further support is provided by a recent study on the bush Karoo rat, in which we showed that individuals with larger home ranges have higher RMR [68]. On the other hand, the absence of a direct link between personality and RMR (or MR after exposure to acute stress), as concluded by our path analysis, would align with the ‘independent model’. However, as argued in [4], even if RMR and personality traits are not directly related but are both driven by an underlying factor, such as by a shared hormonal basis [4], this should not be a valid reason to disregard this correlation. We suggest that this may also be a valid argument in our case. Even indirect associations between metabolism and behavioural traits should remain of interest as long as the neurophysiological mechanisms linking seasonal early life conditions to covarying individual differences in RMR and personality are still poorly understood.

Nevertheless, our study highlights that ignoring important life-history parameters, such as the date of birth within the breeding season, might cause a statistically significant correlation between personality and RMR, which is not meaningful as no causal relationship may exist. This aligns with the main conclusion of a review study [69], which argues that treating MR as a universal pacemaker of biological processes has several major limitations, because it is mainly supported by indirect and correlational evidence. Taken together, our results help explain the inconsistent findings regarding the association between behavioural traits and MR [7], as the relationship may be a context-specific manifestation.

Apart from RMR, we also tested additional metabolic variables that reflect the acute stress response. We observed a temporary fluctuation in MR shortly after acute stress, resembling the ‘peak-decay’ pattern commonly seen in various aspects of the stress response [70]. Although the repeatability of metabolic stress response is only partly supported by short-term data, we found the MR of later-borns increased to a lesser extent after acute stress (relative to their own RMR levels) compared to earlier-borns. The reasons could be that individuals born later in the season experience both higher population density and decreased resource availability [24], i.e. they have to put more effort into foraging and searching for resources. Thus, later-borns might be more habituated to stressful situations due to higher exploring activity, or simply have less energy available to actively respond when encountering stressful situations. Consequently, both their proactive personality and their reduced metabolic stress response can be seen as independent adaptations to being born into a more competitive environment. Similar to RMR, the link between personality and MR stress response is not direct, but independently associated with date of birth.

Individuals born early or late in the breeding season encounter markedly different ecological conditions, which can produce distinct life-history trajectories, especially in short-lived species [24]. The date of birth can represent various aspects of the ecological and social environment during an individual’s early life, influencing both physiological and behavioural development [71,72]. For instance, European shag (*Phalacrocorax aristotelis*) chicks that hatched earlier in the breeding season exhibited greater levels of aggression compared to those hatched later [73]. Humans born in summer have higher basal MR than those born in early winter [74]. We suggest that in bush Karoo rats, later-borns adopt a more proactive personality while maintaining a higher RMR. This could enable them to search and compete for stick lodges (i.e. shelters) more efficiently, particularly as later-borns typically face a more scarce availability of stick lodges than early borns [24].

## 5. Conclusions

Our findings suggest that the date of birth within the breeding season plays a crucial role in shaping both personality and metabolic traits in seasonal breeding female bush Karoo rats. Individuals born later in the breeding season exhibited more proactive personality, higher RMR and lower metabolic responses to acute stress. However, when controlling for the date of birth, there was no significant association between personality and MR, suggesting no direct causal relationship. Instead, our results suggest that the date of birth may act as a shared underlying factor of between-individual variation in MR and behaviour in this species. These findings might help explain why previous studies have reported contradictory results regarding the relationship between MR and personality in other species (reviewed in [5,7]): personality–RMR associations could be driven by shared life-history factors, which are likely context-specific. Overall, our study underscores the importance of considering factors that significantly impact life-history trajectories when investigating the links between energy metabolism and animal personality.

## Data Availability

All data generated or analysed during this study are included in this article and the supplementary material [76].
